# The time is now to use the tools we have to end AIDS in children

**DOI:** 10.1002/jia2.70055

**Published:** 2025-11-14

**Authors:** Lara Vojnov, Roger L. Shapiro, Lloyd B. Mulenga, Adeodata R. Kikitiinwa, Alexandra C. Vrazo, Patricia Fassinou Ekouévi, Nelly Pato Dindi, Appolinaire Tiam, Deborah Persaud, Lynne Mofenson

**Affiliations:** ^1^ Global Health Impact Group Atlanta Georgia USA; ^2^ Department of Immunology and Infectious Diseases Harvard T H Chan School of Public Health Boston Massachusetts USA; ^3^ Zambian Ministry of Health Lusaka Zambia; ^4^ Baylor College of Medicine Children's Foundation Kampala Uganda; ^5^ United States Agency for International Development Washington DC USA; ^6^ Elizabeth Glaser Pediatric AIDS Foundation Abidjan Cote d'Ivoire; ^7^ National AIDS & STI Control Programme (NASCOP) Ministry of Health Nairobi Kenya; ^8^ Milken Institute School of Public Health Department of Epidemiology and Biostatistics George Washington University Washington DC USA; ^9^ Elizabeth Glaser Pediatric AIDS Foundation Washington DC USA; ^10^ Division of Infectious Diseases Department of Pediatrics Johns Hopkins University of School of Medicine Baltimore Maryland USA; ^11^ Department of Molecular Microbiology and Immunology Johns Hopkins Bloomberg School of Public Health Baltimore Maryland USA

**Keywords:** paediatric HIV, point‐of‐care, birth testing, infant diagnosis, treatment, last mile, innovations

## Abstract

**Introduction:**

Progress in reducing and eliminating vertical transmission of HIV in children has stagnated over recent years. Unfortunately, recent decisions in the global donor space are likely to lead to considerably lower funding available for HIV and other disease programmes in high‐burden low‐ and middle‐income settings. Understanding and implementing the most effective strategies to prevent, diagnose, treat and monitor HIV acquisition in children are critical to maximize available resources and provide the best care for children.

**Discussion:**

A set of tools to support the elimination of vertical transmission and end AIDS in children exists across the cascade of care for pregnant mothers and their children, including those for prevention, diagnosis, treatment, monitoring and service delivery. Each tool comes with its own challenges in implementation and scale‐up; however, the effectiveness of many has been well‐studied and documented. Ensuring all women living with HIV have consistent and secure access to optimized antiretroviral therapy will prevent the largest number of leaks in the cascade that can lead to vertical transmission events. Implementing effective infant diagnosis strategies, including same‐day point‐of‐care testing and birth testing where feasible, will lead to earlier and faster identification of children living with HIV. Subsequently, rapid linkage to optimized treatment will save and improve the lives of children living with HIV. Finally, implementing accompanying monitoring modalities and country‐specific service delivery approaches will improve implementation and the effectiveness of these tools as well as retention and commitment in care programmes by children as they become adolescents and adults.

**Conclusions:**

Ending AIDS in children is possible. We have the tools to do so. However, now is the time for national, regional and global stakeholders to come together to prioritize children and ensure appropriate funding, implementation and equitable access are in place. Testing infants quickly and accurately for HIV is the first step to ensuring their lifelong health and wellbeing. As national and global public health structures shift, programmes need to act quickly—gains in paediatric HIV cannot be lost but must be accelerated. Doing so will require political will, financing, infrastructure, community‐led initiatives, and commitment by and access to all to achieve those goals.

## 1 INTRODUCTION

In 2024, 75,000 children died from HIV, while an additional 120,000 acquired HIV [1]. Though scale‐up of infant diagnosis and treatment has improved over the years, the proportion of children living with HIV on lifesaving antiretroviral therapy (ART) has remained stagnant over the past 5 years, with just over half receiving ART [2]. Unfortunately, there has been a clear and consistent gap in the UNAIDS’ 95‐95‐95 targets for children, as compared to adults.

Several global political efforts have been initiated over the past 5 years to galvanize the global, regional, national and subnational communities to work collaboratively to improve the lives and livelihoods of children living with HIV, with the primary goal being to end AIDS. These include the Global Alliance to End AIDS in Children [3] and Paediatric HIV and TB Rome Action Plan [4]. However, progress has been limited. The gaps in diagnosis and treatment coverage for children (treatment coverage: 57%) have remained consistently and substantially lower than those for adults (treatment coverage: 77%) [2]. Additionally, recent decisions by the United States government have led to significant funding losses that have considerably impacted people living with HIV (PWH), their communities, national healthcare systems and programmes, and local, regional and global stakeholders tasked with supporting global and national public health responses [5, 6, 7]. It is estimated that over 7000 children have lost access to ART and nearly 1500 babies have acquired HIV each day (compared to ∼330) since the first stop work orders to United States Agency for International Development‐funded projects were implemented on 24 January 2025 [7].

## 2 DISCUSSION

Since 2010, the World Health Organization has developed a series of recommendations and guidelines for the prevention, diagnosis, treatment and monitoring of children affected or living with HIV [8]. Many of these guidelines have been adapted and adopted by national programmes to reduce vertical transmission, identify children living with HIV earlier, link children living with HIV to lifesaving ART and monitor children for drug resistance. In November 2024, Cepheid (Inc., a subsidiary of Danaher Corporation) and the Elizabeth Glaser Pediatric AIDS Foundation (EGPAF) convened an expert dialogue to discuss existing tools and prevailing challenges in achieving national and global goals for the elimination of HIV in children in an ever‐changing environment. Some of the contents of this article are based on that dialogue.

### 2.1 Prevention of vertical transmission

Prevention of vertical transmission policy and practice has evolved considerably with improved scientific understanding and technological advancements and innovations. After initial focus on non‐pharmacological interventions (e.g. formula‐feeding, caesarean sections, etc.), WHO guidelines have incrementally progressed to now recommend all PWH, including pregnant women, receive lifelong ART, regardless of CD4 count [8]. The preferred, optimized ART regimen includes dolutegravir, a second‐generation integrase strand transfer inhibitor that has a high barrier to drug resistance, high antiviral potency, low pill burden and higher tolerability [9]. These attributes better support pregnant women living with HIV to achieve and maintain viral suppression—significantly reducing their risk of transmitting HIV to their infant [10, 11, 12, 13]. Additionally, infants exposed to HIV are recommended to receive daily prophylaxis (zidovudine and nevirapine or nevirapine alone) for the first 6 weeks of life [8]. New highly effective antiretroviral (ARV) drugs are now available for pre‐exposure prophylaxis (PrEP) in women at risk of HIV, including cabotegravir and lenacapavir, which can be given during pregnancy and lactation [14]. National PrEP rollout can significantly impact HIV seroconversion in pregnant and lactating women, and hence substantially contribute to the prevention of vertical transmission [14]. Finally, national HIV programmes continue to implement and improve identification of women living with HIV through earlier and more frequent HIV testing during pregnancy and postnatally [15]. There has been a rapid uptake and implementation of these guidelines, resulting in considerable impact. Settings with high rates of facility attendance and ART coverage have seen vertical transmission drop as low as <2% [16, 17, 18]. Several countries have nearly eliminated vertical transmission altogether [19].

Although there have been significant gains and the necessary tools exist, full engagement with and implementation of these tools is still lacking in many countries. In particular, incomplete maternal ART coverage from conception to cessation of breastfeeding leads to higher rates of vertical transmission [20]. Additionally, PrEP delivery in antenatal and postnatal care clinics in settings with a high HIV prevalence remains suboptimal [21]. Gaps in the prevention of vertical HIV transmission (PVT) cascade remain, including late and limited antenatal attendance, inconsistent access to testing, treatment, and PrEP, suboptimal rates of healthcare facility deliveries, and poor retention and ineffective engagement models during the postnatal phase—including the imperfect provision of infant post‐natal prophylaxis (PNP). All of these require commitment from the health system, mothers and their families.

### 2.2 Diagnosis of infant HIV infection

Over the last few years, two key tools have become available and are recommended by WHO to rapidly diagnose and treat infants living with HIV: same‐day point‐of‐care testing and birth testing [8]. At least two high‐quality, WHO‐listed, nucleic acid tests for testing infants at the point‐of‐care are available [22]. The diagnostic accuracy of these technologies was greater than 98% sensitive and 99% specific [23]. More importantly, however, a systematic review published in 2022 found that, when tested using a point‐of‐care technology that provides same‐day testing and results, children living with HIV were nearly nine times more likely to start ART within 60 days compared to laboratory‐based testing [24]. This was likely due to the long turnaround times observed with laboratory‐based testing (35 days compared to 0 days with same‐day point‐of‐care testing). Furthermore, same‐day point‐of‐care testing was found to be cost‐saving compared to the standard of care when integrating or sharing platforms across diseases [25].

Nearly a third of vertical transmission happens in utero [26], and children who acquire HIV perinatally are known to have higher mortality rates and more rapid disease progression [27, 28]. Birth testing, with prompt ART initiation, provides an opportunity to reduce early mortality and morbidity among this highly vulnerable population and remains a conditional recommendation by the WHO [8]. Birth testing programmes also support linkage of infants who test negative to the appropriate PNP. Widespread implementation of birth testing has been limited, however, in part due to significant system issues, such as deliveries away from the mother's ART care facility, poor (electronic) linkage of mother‐infant pairs, limited human resource capacity and clinical, practical training to add infant diagnosis to busy maternity wards, and rapid discharge of healthy mothers and newborns before results can be delivered.

Unfortunately, despite the clear clinical, ethical and cost benefits of these two interventions, there has been limited implementation. Laboratory‐based testing has prevailed in nearly all high‐burden countries as the predominant technology used to diagnose children living with HIV, resulting in long turnaround times and considerable missed opportunities. Inadequate early infant diagnosis testing coverage continues, with <70% of children tested before 6 weeks of life [1]. Cost plays a role in some of these decisions. Same‐day point‐of‐care infant diagnosis has remained cost‐prohibitive and unaffordable in many low‐ and middle‐income country (LMIC) settings, with the cost per test exceeding $14 in nearly all instances. Resource scarcity (both actual and perceived) may negatively impact point‐of‐care technology implementation and placement with devices placed in hub or distant laboratory sites, negating the clinical benefits of these technologies. Careful mapping of existing technologies and targeted placement and decentralization can improve immediate access and result utilization, all while technological innovations continue for simpler and cheaper tests.

Finally, as vertical transmission rates remain low in some settings, the challenge of maintaining test performance increases as incidence declines. Lowering incidence rates results in declining positive predictive values that lead to false positive test results [29]. Additionally, indeterminate and false negative results remain a diagnostic challenge [30, 31]. These issues will require global and national policymakers to work together to develop more efficient and effective testing, particularly by updating and revising infant diagnosis algorithms, ensuring testing throughout and until the cessation of breastfeeding, implementing tests with high performance, and ensuring that all positive tests are confirmed with a second sample to guarantee that only children living with HIV are linked to lifelong treatment.

### 2.3 Treatment in children

Until recently, treatment tools and access to ART were relatively limited—both for children and adults. However, with the introduction of TLD (tenofovir‐lamivudine‐dolutegravir (DTG)) for all PWH, realizing the prevention benefits of ART, including for vertical transmission, is now a reality. For children, transition to optimized treatment has lagged behind that for adults. For too long, ART for children was marked by limited options, bad‐tasting syrups that were poorly tolerated and dosing issues. Fortunately, optimized ARVs are now available for children that have improved tolerability, fewer side effects, more palatable formulations, a higher barrier to drug resistance and once‐daily dosing that allow for much more simplified lifelong ART. In fact, most children are now on a DTG‐based regimen with over 95 countries procuring paediatric DTG (pDTG) [32]. Additionally, paediatric ALD (pALD: abacavir [ABC]/lamivudine [3TC]/DTG), a once‐daily, fixed‐dose pill, is now available and being implemented in over 10 countries, and additional ARVs continue to be investigated [32].

The development and introduction of pDTG and pALD are likely to lead to significant improvements in the lives of children living with HIV, particularly lives saved, better management and adherence to treatment, and increased viral suppression [33, 34]. However, broader scale‐up and consistent, long‐term access are required. At present, pDTG can only be given to children 4 weeks of age or weighing at least 3 kilograms; however, emerging evidence supports the extension of pDTG dosing starting at birth [35, 36]. Similarly, pALD is currently only available for children 3 months of age or weighing at least 6 kilograms [32]; however, a recent study suggests that pALD can be used from 4 weeks of age [37]. Further research and development are required to develop optimized treatments for younger children and for children who experience adverse effects with DTG‐based ART. Ideally, long‐acting treatments, including ARVs, broadly neutralizing antibodies or another long‐term treatment derivative, would be developed to support more effective treatment strategies for all children living with HIV. Lastly, additional research and understanding into the long‐term health consequences, including the mental health, of children living with HIV, as well as HIV‐negative children with perinatal HIV exposure, may provide opportunities to better support their lifelong health and wellbeing.

### 2.4 Monitoring children on treatment

Continual monitoring of children living with HIV and taking an optimized ART regimen is critical to ensure that their drug regimen continues to be efficacious, especially because of their prior exposure to ARVs used for infant prophylaxis of vertical transmission. This primarily includes HIV viral load testing, but should also consider additional monitoring tools, such as CD4, to identify advanced HIV disease, tuberculosis screening and diagnosis, and other infectious disease screenings and testing, depending on prevalence rates where the children reside. With the introduction and scale‐up of optimized drug regimens, approximately 86% of children living with HIV and on ART had a suppressed viral load [38]. Access to viral load testing has progressively increased since initial widescale introductions began in 2011. Though nearly 30 million viral load tests were run in 2023 [32], coverage needs to further expand, particularly to reach all children living with HIV and on ART.

Most viral load testing to date is performed using laboratory‐based molecular technologies [22]. The preferred sample type for most assays is plasma separated from whole blood; however, the blood sample generally needs to reach the laboratory within 24–48 hours of collection to ensure high‐quality testing [39]. This can be challenging in many LMIC settings. Alternative sample types, such as dried blood spot samples and plasma separation cards, have been developed and can support widespread access to this important monitoring test. Additionally, (near) point‐of‐care molecular assays exist on the market and allow national programmes to increase access to testing, including for both infant diagnosis as well as viral load monitoring for both the infant and mother, expeditiously and simultaneously, if needed.

Several challenges remain in the widespread implementation and use of viral load testing to support the monitoring of children living with HIV. Laboratory‐based testing tends to be fragmented, complicated and long—sample transportation and supply chain logistics are often challenging, and test results can take 3–6 weeks to be returned to the clinician and caregiver [39, 40]. Although dried blood spot samples can be stored and shipped over the course of days and weeks instead of hours and can be prepared using freshly collected blood, dried blood spot samples do not have ideal performance and could be improved to support wider implementation [41]. Plasma separation cards also allow for considerably longer storage and shipment; however, plasma must be separated on site before application to the card, significantly limiting decentralization as centrifugation is required [39]. Finally, though near point‐of‐care technologies can substantially reduce the time to test result receipt and utilization, implementation has been limited, primarily due to high costs. As such, even though the tools exist and have been on the market for several years, decentralization of and widespread rapid access to viral load testing has been severely limited, including for high‐priority populations such as pregnant women and children.

### 2.5 Service delivery

The primary way to end AIDS in children is to ensure all women living with HIV are identified and on optimized ART throughout any pregnancies and breastfeeding. This will prevent nearly all vertical transmission events, resulting in minimal need for infant testing, treatment and monitoring. However, we are far from that reality.

While optimizing programmes to provide ART to women throughout the vertical transmission cascade, we also need to focus attention on opportunities to reach children. Nearly all children present to healthcare facilities in their first 2 years of life (if not earlier), whether through vaccination, well baby visits or sick visits; however, the testing coverage for infant diagnosis has stagnated as programmes primarily focus on testing children of women registered in PVT programmes. A myriad of other case‐finding opportunities exist that are consistently poorly implemented, such as index and family testing that includes the children and sick child visits (e.g. nutrition, tuberculosis and inpatient wards)—determining the exposure and acquisition status of children in these scenarios could significantly increase case detection rates [8]. Further, testing and diagnosis through the full period of transmission risk, including and especially end‐of‐exposure testing (i.e. after breastfeeding cessation), is not commonly implemented yet critical to support the identification of transmission events occurring during breastfeeding [42]. Infant HIV diagnosis should be seen as a multi‐step process from which children might only “graduate” at the end of the exposure period, not with a negative test at 6 weeks of age.

Once children living with HIV are identified, in addition to the necessary optimized treatment and monitoring tools, continuous provision of key service delivery interventions will support their retention in care. These include peer‐supported community‐based HIV care delivery and mental health models [43, 44]. Peer‐supported community‐based HIV care delivery models include community health workers, peer mentors and support groups that can support the children, adolescents and/or caregivers as they navigate the healthcare system, identify helpful community resources and provide psychosocial support. These models have been shown to improve HIV viral suppression and retention in care [43, 44].

Finally, consistent access to prophylaxis and treatment is critical to see the benefits of these tools impact all populations. Access can be interrupted by drug stock‐outs, incorrect drug regimens prescribed, stigma and discrimination, and poor adherence. Addressing these challenges requires a myriad of stakeholders to commit to long‐term provision and engagement with all facets of national HIV programmes: strong procurement practices; streamlined supply chains; trained, compassionate medical personnel free from stigma; and a variety of implementation models that suit each child, adolescent and adult living with HIV. The existing, optimized tools described here, coupled with these critical service delivery interventions, allow for PWH to fully participate in their care to live long and healthy lives.

## 3 CONCLUSIONS

As countries revise their priorities, plans and long‐term strategies to develop more sustainably financed health and national HIV programmes, the elimination of vertical HIV transmission requires consistent prioritization. The tools to end AIDS in children exist and should be adapted to implement the most effective country‐specific combinations. In addition to the provision of maternal ART throughout pregnancy and breastfeeding, early and frequent testing of infants exposed to HIV is a critical step in the pathway to ending AIDS in children. Closing the remaining gaps in PVT will be incredibly impactful, while implementing broader case‐finding strategies, including birth testing where feasible and same‐day point‐of‐care technologies, will improve earlier and quicker identification of children living with HIV. Scaling up innovative, optimized treatment will save and improve the lives of children living with HIV, while improving monitoring and service delivery approaches will ensure not just survival, but long and healthy lives.

## COMPETING INTERESTS

LM/AT: ViiV provided funding to EGPAF for a pilot birth surveillance study in Eswatini, 2021–2023. Cepheid funded EGPAF for a now‐completed study using the GeneXpert. The remaining authors declare no competing interests.

## AUTHOR CONTRIBUTIONS

LV: Attended and contributed to the dialogue; conceived of and drafted the initial manuscript, revised and finalized for submission; LM, RLS, LBM, ARK, ACV, PFE, NPD, AT and DP: Attended and contributed to the dialogue; reviewed the manuscript and approved of submission.

## Data Availability

Data sharing is not applicable to this article, as no datasets were generated or analysed during the current study.

## References

[1] UNAIDS . Global AIDS Update 2025: AIDS, crisis and the power to transform. 2025. (https://www.unaids.org/en/resources/documents/2025/2025‐global‐aids‐update, accessed 20 August 2025).

[2] Mutanga JN , Ronan A , Powis KM . Achieving equity for children and adolescents with perinatal HIV exposure: an urgent need for a paradigm shift. J Int AIDS Soc. 2023;26(Suppl 4):e26171. 37909238 10.1002/jia2.26171 PMC10618885

[3] UNAIDS . The Global Alliance to End AIDS in Children by 2030. (https://www.unaids.org/sites/default/files/media_asset/global‐alliance‐end‐AIDS‐in‐children_en.pdf, accessed 11 April 2025).

[4] Paediatric HIV and TB: Rome Action Plan. (https://www.paediatrichivactionplan.org, accessed 11 April 2025).

[5] Lankiewicz E , Sharp A , Drake P , Sherwood J , Macharia B , Ighodaro M , et al. Early impacts of the PEPFAR stop‐work order: a rapid assessment. J Int AIDS Soc. 2025;28(2):e26423. 39964153 10.1002/jia2.26423 PMC11834162

[6] Nichols BE , Geng EH , Moakley E , Phillips AN , Imai‐Eaton JW , Stover J , et al. Rapid development of an online tracker to communicate the human impact of abruptly halting PEPFAR support. J Int AIDS Soc. 2025;28(3):e26433. 40012206 10.1002/jia2.26433 PMC11865335

[7] amfAR . Impact of stop work orders for PEPFAR programs. 2025. (https://www.amfar.org/wp‐content/uploads/2025/01/Impact‐of‐Stop‐Work‐Orders‐for‐PEPFAR‐Programs‐2.pdf, accessed 11 April 2025).

[8] World Health Organization . Consolidated guidelines on HIV prevention, testing, treatment, service delivery and monitoring: recommendations for a public health approach. 2021. (https://www.who.int/publications/i/item/9789240031593, accessed 11 April 2025). 34370423

[9] Kandel CE , Walmsley SL . Dolutegravir—a review of the pharmacology, efficacy, and safety in the treatment of HIV. Drug Des Dev Ther. 2015;9:3547–3555. 10.2147/DDDT.S84850 PMC4500604 26185421

[10] Gill MM , Hoffman HJ , Ndatimana D , Mugwaneza P , Guay L , Ndayisaba GF , et al. 24‐month HIV‐free survival among infants born to HIV‐positive women enrolled in Option B+ program in Kigali, Rwanda: the Kabeho study. Medicine (Baltimore). 2017;96(51):e9445. 29390577 10.1097/MD.0000000000009445 PMC5758279

[11] Myer L , Phillips TK , McIntyre JA , Hsiao N‐Y , Petro G , Zerbe A , et al. HIV viraemia and mother‐to‐child transmission risk after antiretroviral therapy initiation in pregnancy in Cape Town, South Africa. HIV Med. 2017;18(2):80–88. 27353189 10.1111/hiv.12397

[12] Townsend CL , Byrne L , Cortina‐Borja M , Thorne C , de Ruiter A , Lyall H , et al. Earlier initiation of ART and further decline in mother‐to‐child HIV transmission rates, 2000–2011. AIDS. 2014;28(7):1049–57. 24566097 10.1097/QAD.0000000000000212

[13] Walters MK , Bulterys MA , Barry M , Hicks S , Richey A , Sabin M , et al. Probability of vertical HIV transmission: a systematic review and meta‐regression. Lancet HIV. 2025;12(9):e638–e648. 40753992 10.1016/S2352-3018(25)00132-8 PMC12720210

[14] World Health Organization . Guidelines on lenacapavir for HIV prevention and testing strategies for long‐acting injectable pre‐exposure prophylaxis. 2025. (https://www.who.int/publications/i/item/9789240111608, accessed 20 August 2025). 40743386

[15] World Health Organization . Consolidated guidelines on differentiated HIV testing services. 2024. (https://iris.who.int/bitstream/handle/10665/378162/9789240096394‐eng.pdf?sequence=1, accessed 11 April 2025). 39116265

[16] Kesho Bora Study Group, de Vincenzi I . Triple antiretroviral compared with zidovudine and single‐dose nevirapine prophylaxis during pregnancy and breastfeeding for prevention of mother‐to‐child transmission of HIV‐1 (Kesho Bora study): a randomised controlled trial. Lancet Infect Dis. 2011;1(3):171–80. 10.1016/S1473-3099(10)70288-7 21237718

[17] Shapiro RL , Hughes MD , Ogwu A , Kitch D , Lockman S , Moffat C , et al. Antiretroviral regimens in pregnancy and breast‐feeding in Botswana. N Engl J Med. 2010;362(24):2282. 20554983 10.1056/NEJMoa0907736 PMC2999916

[18] Flynn PM , Taha TE , Cababasay M , Fowler MG , Mofenson LM , Owor M , et al. Prevention of HIV‐1 transmission through breastfeeding: efficacy and safety of maternal antiretroviral therapy versus infant nevirapine prophylaxis for duration of breastfeeding in HIV‐1‐infected women with high CD4 cell count (IMPAACT PROMISE): a randomized, open label, clinical trial. J Acquir Immune Defic Syndr. 2018;77(4):383. 29239901 10.1097/QAI.0000000000001612 PMC5825265

[19] The path to elimination of vertical transmission of HIV. Lancet HIV. 2022;9(2):e67. 35120634 10.1016/S2352-3018(22)00012-1

[20] UNAIDS epidemiological estimates. 2023. (https://aidsinfo.unaids.org, accessed 11 April 2025).

[21] Davey DLJ , Mvududu R , Mashele N , Bheemraj K , Khadka N , Johnson LF , et al. Initiation and continued use of oral pre‐exposure prophylaxis among pregnant and postpartum women in South Africa (PrEP‐PP): a demonstration cohort study. Lancet HIV. 2024;11(11):e746–e755. 39477557 10.1016/S2352-3018(24)00240-6 PMC13444643

[22] World Health Organization . List of prequalified in vitro diagnostic products. 2025. (https://extranet.who.int/prequal/vitro‐diagnostics/prequalified‐vitro‐diagnostics, accessed 11 April 2025).

[23] Ochodo EA , Kakourou A , Mallett S , Deeks JJ . Point‐of‐care tests detecting HIV nucleic acids for diagnosis of HIV infection in infants and children aged 18 months or less. Cochrane Database Syst Rev. 2018;2018(11):CD013207. 10.1002/14651858.CD013207.pub2 PMC8406580 34383961

[24] Luo R , Fong Y , Boeras D , Jani I , Vojnov L . The clinical effect of point‐of‐care HIV diagnosis in infants: a systematic review and meta‐analysis. Lancet. 2022;400(10356):887–895. 36116479 10.1016/S0140-6736(22)01492-1

[25] Le Roux SM , Odayar J , Sutcliffe CG , Salvatore PP , de Broucker G , Dowdy D , et al. Cost‐effectiveness of point‐of‐care versus centralized, laboratory‐based nucleic acid testing for diagnosis of HIV in infants: a systematic review of modelling studies. Lancet HIV. 2023;10(5):e320–331. 37149292 10.1016/S2352-3018(23)00029-2 PMC10175481

[26] Amin O , Powers J , Bricker KM , Chahroudi A . Understanding viral and immune interplay during vertical transmission of HIV: implications for cure. Front Immunol. 2021;12:757400. 34745130 10.3389/fimmu.2021.757400 PMC8566974

[27] Becquet R , Marston M , Dabis F , Moulton LH , Gray G , Coovadia HM , et al. Children who acquire HIV infection perinatally are at higher risk of early death than those acquiring infection through breastmilk: a meta‐analysis. PLoS One. 2012;7:e28510. 22383946 10.1371/journal.pone.0028510 PMC3285615

[28] Marston M , Becquet R , Zaba B , Moulton LH , Gray G , Coovadia H , et al. Net survival of perinatally and postnatally HIV‐infected children: a pooled analysis of individual data from sub‐Saharan Africa. Int J Epidemiol. 2011;40:385–396. 21247884 10.1093/ije/dyq255 PMC3140269

[29] Monaghan TF , Rahman SN , Agudelo CW , Wein AJ , Lazar JM , Everaert K , et al. Foundational statistical principles in medical research: sensitivity, specificity, positive predictive value, and negative predictive value. Medicina (Mex). 2021;57(5):503. 10.3390/medicina57050503 PMC8156826 34065637

[30] World Health Organization . Update on antiretroviral regimens for treating and preventing HIV infection and update on early infant diagnosis of HIV: interim guidance. 2018. (https://iris.who.int/bitstream/handle/10665/273129/WHO‐CDS‐HIV‐18.19‐eng.pdf, accessed 20 Aug 2025).

[31] Radebe L , Haeri Mazanderani A , Sherman GG . Indeterminate HIV PCR results within South Africa's early infant diagnosis programme, 2010–2019. Clin Microbiol Infect. 2022;28(4):609.e7–609.e13. 10.1016/j.cmi.2021.08.002 34400341

[32] Clinton Health Access Initiative . 2024 HIV Market Report. Issue 15, December 2025. (https://www.clintonhealthaccess.org/wp‐content/uploads/2024/12/2024‐HIV‐Market‐Report_12.20.24.pdf, accessed 11 April 2025).

[33] Amuge P , Lugemwa A , Wynne B , Mujuru HA , Violari A , Kityo CM , et al. Once‐daily dolutegravir‐based antiretroviral therapy in infants and children living with HIV from age 4 weeks: results from the below 14 kg cohort in the randomised ODYSSEY trial. Lancet HIV. 2022;9(9):e638–e648. 36055295 10.1016/S2352-3018(22)00163-1 PMC9646993

[34] Bacha JM , Dlamini S , Anabwani F , Gwimile J , Kanywa JB , Farirai J , et al. Realizing the promise of dolutegravir in effectively treating children and adolescents living with HIV in real‐world settings in 6 countries in Eastern and Southern Africa. Pediatr Infect Dis J. 2023;42(7):576–581. 36795586 10.1097/INF.0000000000003878 PMC10259212

[35] Bekker A . Multidose PK/safety of dolutegravir dispersible tablets and oral films in neonates: PETITE‐DTG study. Conference on Retroviruses and Opportunistic Infections. San Francisco, USA. 2025. Abstract 122.

[36] Panjasawatwong N , Bekker A , Rabie H , du Toit S , Than‐in‐at K , Groenewald M , et al. Alternative dosing of ABC/3TC dispersible tablets to align with dolutegravir dosing in neonates. Conference on Retroviruses and Opportunistic Infections. San Francisco, USA. 2025. Abstract 1048.

[37] Chandasana H , Brooks KM , Buchanan AM , Tan L , Mujuru H , Colbers A , et al. A single once daily ABC/DTG/3TC tablet predicts safe and effective exposures in children 3 to <6kg. Conference on Retroviruses and Opportunistic Infections. San Francisco, USA. 2025. Abstract 944.

[38] UNAIDS . Fact sheet. 2024. (https://www.unaids.org/sites/default/files/media_asset/UNAIDS_FactSheet_en.pdf, accessed 11 April 2025).

[39] World Health Organization . HIV molecular diagnostics toolkit to improve access to viral load testing and infant diagnosis. 2019. (https://www.who.int/publications/i/item/9789241516211, accessed 11 April 2025).

[40] World Health Organization . Updated recommendations on HIV prevention, infant diagnosis, antiretroviral initiation and monitoring. 2021. (https://www.who.int/publications/i/item/9789240022232, accessed 11 April 2025). 33822559

[41] Vojnov L , Carmona S , Zeh C , Markby J , Boeras D , Prescott MR , et al. The performance of using dried blood spot specimens for HIV‐1 viral load testing: a systematic review and meta‐analysis. PLoS Med. 2022;19(8):e1004076. 35994520 10.1371/journal.pmed.1004076 PMC9447868

[42] Thomson KA , Hughes J , Baeten JM , John‐Stewart G , Celum C , Cohen CR , et al. Increased risk of HIV acquisition among women throughout pregnancy and during the postpartum period: a prospective per‐coital‐act analysis among women with HIV‐infected partners. J Infect Dis. 2018;218:16–25. 29514254 10.1093/infdis/jiy113 PMC5989601

[43] Mavhu W , Willis N , Mufuka J , Bernays S , Tshuma M , Mangenah C , et al. Effect of a differentiated service delivery model on virological failure in adolescents with HIV in Zimbabwe (Zvandiri): a cluster‐randomised controlled trial. Lancet Glob Health. 2020;8(2):e264–275. 31924539 10.1016/S2214-109X(19)30526-1

[44] Simms V , Weiss HA , Chinoda S , Mutsinze A , Bernays S , Verhey R , et al. Peer‐led counselling with problem discussion therapy for adolescents living with HIV in Zimbabwe: a cluster‐randomised trial. PLoS Med. 2022;19(1):e1003887. 34986170 10.1371/journal.pmed.1003887 PMC8730396

